# Complete Genome Sequences of Two Temperate Bacillus subtilis Phages Isolated at Tumamoc Hill Desert Laboratory

**DOI:** 10.1128/mra.00455-22

**Published:** 2022-08-17

**Authors:** Greg P. Krukonis, Amanda K. Kemp, Katie F. Storrie, Vivian R. Chavira, Hayden W. Lantrip, Victoria D. Perez, Desiree A. Reyes, Julian A. Truax, Rachel Loney, Véronique A. Delesalle

**Affiliations:** a Department of Biology, Angelo State University, San Angelo, Texas, USA; b Department of Biology, Gettysburg College, Gettysburg, Pennsylvania, USA; DOE Joint Genome Institute

## Abstract

Bacteriophages are important in structuring bacterial communities, including desert soils dominated by *Bacillus* species. Here, we describe two genetically similar temperate phages isolated on a Bacillus subtilis strain from soil in Tucson, Arizona. Their double-stranded DNA (dsDNA) genomes contain 98 and 102 genes, with a set of 4 genes being found in only one phage.

## ANNOUNCEMENT

Bacterial communities in desert environments are often dominated by *Firmicutes* strains, including Bacillus subtilis and relatives (1–4). Given how bacteriophages impact bacterial communities (5–7), understanding these communities requires understanding phage diversity. Here, we describe two temperate phages from the Sonoran Desert.

Each phage was isolated from its own soil sample collected at Tumamoc Hill Desert Laboratory (Tucson, AZ) in May 2016 (32°13′04.9″N, 111°00′12.9″W), at sites separated by 10 m. The soil was dry and sandy, dug to 10 cm. Approximately 1 g of soil was added to 20 mL LB broth, incubated for 4 h at 37°C with shaking at 250 rpm, and then filtered (0.22 μm). Samples were then plated on Bacillus subtilis strain T89-06 (also called S89-6 or T89-6), which was originally isolated by Istock and colleagues (8, 9). Individual plaques were isolated and were single plaque purified three times on lawns made from spores of the isolation host. High-titer lysates were prepared by flooding, with LB broth, multiple plates containing at least 10^4^ plaques. Lysates were filtered, and DNA was extracted using phenol-chloroform (10). For sequencing, libraries were prepared with the Illumina TruSeq Nano DNA library preparation kit and sequenced with the Illumina MiSeq platform, using a 150-bp single-end read v3 flow cell, at the North Carolina State University Genomic Science Laboratory. We assembled genomes using GS De Novo Assembler v2.9 (11). For each phage, the 150-bp reads were assembled into one contig with >1,000× coverage, and contig consensus quality was verified in Consed v29 (12) (Table 1). Genome ends were determined with PhageTerm (13) (Table 1). The finished sequences were imported into DNA Master v5.22.22 (http://cobamide2.bio.pitt.edu/computer.htm) to map and compare open reading frames. Putative genes were called based on both Glimmer v3.0 and GeneMark v2.5 algorithms (14, 15). Putative protein functions were predicted using BLAST v2.12 (16) and HHpred (17). For BLASTp matches, an E value of <10^−5^ was required to assign function. For HHpred matches, a high probability (>85%), substantial coverage (>50%), and low E value (<10^−5^) were required. The absence of tRNA genes was confirmed with ARAGORN (18). Default settings were used for all programs.

**TABLE 1:** Sequencing information and genome characteristics for *Bacillus* phages 268TH002 and 268TH007

Phage name	No. of reads	Coverage (×)	Genome size (bp)	GC content (%)	Genome ends^*a*^	No. of protein-coding genes	Best BLASTn match (GenBank accession no.)^*b*^	Query coverage (%) with best match	Identity (%) with best match
268TH002	807,065	1,868	65,534	47.3	310-bp DTRs	98	Bacillus velezensis strain Lzh-a42 (CP025308.1)	63	88
							*Bacillus* phage vB_BauS_KLEB27-1 (OM654379.1)	24	76
268TH007	505,753	1,124	68,062	47.4	310-bp DTRs	102	Bacillus velezensis strain Lzh-a42 (CP025308.1)	61	88
							*Bacillus* phage vB_BauS_KLEB27-1 (OM654379.1)	23	76

a DTR, direct terminal repeat. By convention, genomes start and end with the DTR sequence and with the terminase gene on the forward strand (11).

b The genome of each phage was compared to the complete nucleotide database and to the same database restricted to all tailed phages (combined taxid numbers 10699, 10662, and 10744) with BLASTn. For each search, the best match is reported.

Phages 268TH002 and 268TH007 have double-stranded DNA (dsDNA) genomes with 98 and 102 predicted protein coding genes, respectively (Table 1), and a genome organization typical of *Siphoviridae*, with structural genes showing conserved order (19). They show limited nucleotide similarity to other sequenced phages (Table 1) but share 96% nucleotide identity with each other, differing primarily through the presence of 2,525 bp in the middle of the genome of 268TH007 with four open reading frames (putatively coding for a FtsK-like DNA translocase, a replication-relaxation family protein, a helix-turn-helix transcriptional regulator, and a hypothetical protein). FtsK translocases are involved in the bacterial SOS response to DNA damage, can activate prophage induction (20), and may broaden conditions for prophage induction. In addition, two genes whose predicted products have sequence identity to tyrosine recombinase have been identified. Whether and how these function in phage integration are open questions. Finally, both phages have ribonucleotide reductase genes, which may benefit them through synthesis of deoxyribonucleotides during periods when host DNA synthesis is inactive (21).

### Data availability.

Genome sequences and associated information can be found under the following GenBank and SRA accession numbers: 268TH002, ON210835 and SRX15148566; 268TH007, ON210834 and SRX15148567, respectively.
